# A27 ROLE OF HIPPO SIGNALING EFFECTORS YAP & TAZ IN GASTRIC METAPLASIA AND TUMORIGENESIS

**DOI:** 10.1093/jcag/gwad061.027

**Published:** 2024-02-14

**Authors:** Z Ju, M Farag, M Haddad, P Fiset, L Lorenzo, A Gregorieff

**Affiliations:** Pathology, McGill University, Montreal, QC, Canada; McGill University, Montreal, QC, Canada; Pathology, McGill University, Montreal, QC, Canada; Pathology, McGill University, Montreal, QC, Canada; Pathology, McGill University, Montreal, QC, Canada; Pathology, McGill University, Montreal, QC, Canada

## Abstract

NOT PUBLISHED AT AUTHOR’S REQUEST

Please acknowledge all funding agencies listed below

Funding Agencies

CIHR

